# Comparative analysis reveals assortative mate preferences in darters independent of sympatry and sex

**DOI:** 10.1002/ece3.11498

**Published:** 2024-09-29

**Authors:** Yseult Héjja‐Brichard, Julien P. Renoult, Tamra C. Mendelson

**Affiliations:** ^1^ Department of Biological Sciences University of Maryland, Baltimore County Baltimore Maryland USA; ^2^ CEFE, Univ Montpellier, CNRS, EPHE, Univ Paul‐Valery Montpellier Montpellier Occitanie France

**Keywords:** *Etheostoma*, meta‐analysis, sexual isolation, speciation

## Abstract

A preference for mating with conspecifics over heterospecifics is fundamental to the maintenance of species diversity in sexually reproducing organisms. This type of positive assortative preference results in sexual isolation, and a reduction in gene flow between species due to differences in mate choice. The proximate and ultimate causes of sexual isolation therefore constitute active areas of research in evolutionary biology. Sexual isolation is often stronger between closely related sympatric species as compared to allopatric species because of processes such as reinforcement. In addition, traditional theories of sexual selection suggest that because reproduction is more costly to females, they should be the choosier sex and play a more central role in sexual isolation. We conducted a comparative analysis of assortative mate preferences in males and females of sympatric and allopatric species pairs of darters (fish genus *Etheostoma*). We performed a meta‐analysis of 17 studies, encompassing 21 species, in which assortative preference was measured when fish were (in most cases) allowed only visual information. As expected, we found stronger preferences for conspecifics over heterospecifics across studies and species. However, we did not find an effect of sympatry or sex on the strength of preference for conspecifics, but rather remarkable variation across species. We offer several testable hypotheses to explain the variation we observed in the strength of assortative preference.

## Graphical abstract

Mating preference for conspecifics is fundamental to species diversity maintenance in sexually reproducing organisms. Previous comparative analyses suggest that geography plays a key role in such preferences. Our comparison of sympatric and allopatric species of darters (genus *Etheostoma*) revealed that the link between assortative mating and geography is not as straightforward as might be predicted from hypotheses of reinforcement, and the link between assortative mating and sex is not as straightforward as classical interpretations of sexual selection might suggest.

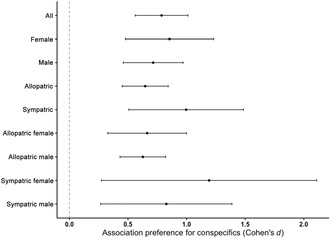

## 1 INTRODUCTION

As evolutionary biology continues to explore the mechanisms of speciation, the processes driving reproductive isolation remain a central focus of study. One important reproductive barrier is sexual isolation, a reduction in gene flow due to differences in mate choice. Sexual isolation is a consequence of assortative mating resulting from a preference for conspecific mates (e.g., Kopp et al., 2018). Preference for conspecific over closely related heterospecific mates (“assortative preference”) can evolve as a result of divergent natural or sexual selection of courtship traits and preferences in geographically isolated populations. Such a preference depends on contexts such as resource acquisition (Hunt et al., 2005) or predation risk (Godin & Briggs, 1996) and varies between species, from a strict preference for conspecifics to a preference for heterospecifics (e.g., in the swordtail fish, Pilakouta et al., 2017; Ryan & Wagner, 1987), and it can be strengthened in sympatric populations due to processes such as reinforcement (Dobzhansky, 1940; Liou & Price, 1994; Servedio & Noor, 2003). Understanding the proximate and ultimate mechanisms driving assortative preference constitutes an active area of research in the field of speciation.

Prezygotic barriers are twice as strong in sympatric species of *Drosophila* as in allopatric species (Coyne & Orr, 1989, 1997). This geographic pattern was not true for postzygotic barriers (hybrid inviability and hybrid sterility). This pattern in *Drosophila* was later confirmed to be a result of mate choice and not gametic incompatibility (Yukilevich, 2012). The same pattern was found at a smaller scale in two frog species of the genus *Pseudacris* (Lemmon, 2009). Females from populations in which the two species were sympatric more strongly preferred conspecific signals, compared with allopatric females, and this preference was stronger when the conspecific signal was the sympatric one (Lemmon, 2009). Comparative studies in plants also support a pattern of greater assortative mating in sympatric species, although (Hopkins, 2013) points out ways in which those studies could be strengthened. These results are often interpreted as evidence for reinforcement when selection against hybrids in sympatry favors increased preference for conspecific mates (Servedio & Noor, 2003). Reinforcement is thought to increase assortative mating or reproductive isolation if there is a genetic basis to mate choice because the alleles that facilitate heterospecific mating decrease in frequency via the reduction in hybrid fitness. Assortative mating can also increase in sympatric species through processes such as differential fusion (Noor, 1999) and species sorting (Kyogoku & Wheatcroft, 2020), which can yield the same pattern of stronger sexual isolation in sympatry (see Matute & Cooper, 2021). Regardless, stronger assortative preference in populations that are sympatric with a close congener is an important pattern that can both support and generate hypotheses about the evolutionary mechanisms driving sexual isolation.

Another important result of broad comparative studies in *Drosophila* is that of Yukilevich and Peterson (2019), who found that for sympatric species pairs, female *Drosophila* had stronger preferences for conspecific males than males did for females, whereas no sex difference in preference was found in allopatric species. That result is consistent with classic sexual selection theory (Andersson, 1994; Darwin, 1871; Trivers, 1972 but see Edward & Chapman, 2011) that females invest more than males in reproduction and thus represent the choosier sex. A greater strength of preference for conspecific mates in females of sympatric populations thus could suggest two things. Mating with a heterospecific in sympatry might be more costly for females than males, leading to reinforcement of female more so than male preferences; or female preferences evolve faster than male preferences in allopatry, and they are necessary for maintaining species boundaries upon secondary contact.

Here, we focused on darters (genus *Etheostoma*) to perform a comparative analysis of preference for conspecific mates. Darters are a large clade of North American freshwater fishes in which males are characterized by elaborate secondary sexual traits (Page & Burr, 2011). We focused on this genus because mate preference and mate choice have now been investigated in numerous pairs of species of darters, making them an emerging model for understanding the role of mate choice in speciation. Studies find that species demonstrate a varying degree of assortative preference (Martin & Mendelson, 2013; Mendelson et al., 2018; Williams & Mendelson, 2013) and that assortative preference and assortative mate choice is present in both females (Roberts et al., 2017; Williams & Mendelson, 2010, 2011) and males (Ciccotto et al., 2013; Martin & Mendelson, 2016; Moran et al., 2017; Moran & Fuller, 2018; Roberts & Mendelson, 2017; Zhou et al., 2015), depending on which heterospecific is presented. Moreover, hybridization has been documented in many darter species (Bossu & Near, 2013; Keck & Near, 2009), which could provide the substrate for reinforcement (i.e., hybrids with reduced fitness), making this clade a good system to examine geographic and sex‐specific patterns of assortative mate preference at the genus level.

Two studies of darters have tested explicitly for stronger assortative preference in sympatric populations in one or a small number of species pairs. Moran and Fuller (2018) compared male choice and aggressive behaviors in a small number of closely related species (*Etheostoma caeruleum* and members of the *Ceasia* species complex). They found that both male preferences for conspecific females and aggressive behaviors of males toward conspecific males were stronger in populations that were sympatric with the congener. Roberts and Mendelson (2020) measured the strength of assortative preference in allopatric and sympatric populations of two darter species (*Etheostoma zonale* and *E. barrenense*). They also found a stronger preference for conspecific mates in sympatric populations, but only in females, which contrasts the results of Moran and Fuller (2018), who found assortative preference only in males. Notably, the experimental design in these two studies differed in whether fish were allowed to physically interact and spawn. *Etheostoma caeruleum* and *Ceasia* species were allowed full access to one another, with males competing physically for females. *Etheostoma zonale and E. barrenense* were presented conspecific and heterospecific mates behind a partition and allowed only visual information; assortative preference was measured by the amount of time a fish spent adjacent to the tank with a conspecific versus a heterospecific.

These two experimental designs represent a trade‐off in the study of mate preference. Allowing physical contact in preference experiments simulates more realistic mating interactions that may better represent interactions and outcomes in nature (also see Moran et al., 2017; Zhou et al., 2015). But allowing physical contact also may mask preferences, if intrasexual (here, male) competition prevents intersexual (here, female) preferences from manifesting as mate choice. To examine the proximate and ultimate causes of assortative preference, an experimental design that allows animals to express their preference without physical interference is critical. In freshwater fishes, dichotomous mate choice designs that measure the amount of time spent associating with one option over another have been shown to reliably represent an individual's preference (Brooks & Endler, 2001; Gonçalves & Oliveira, 2003; Jeswiet & Godin, 2011; Lehtonen & Lindström, 2008; Martin & Mendelson, 2013; Williams & Mendelson, 2010).

Here, we performed a phylogenetically informed meta‐analysis of dichotomous mate choice studies in *Etheostoma* to determine the extent to which sympatry and sex affect the strength of assortative preference. Studies were conducted over the course of 14 years by multiple researchers following similar experimental protocols in the lab of a single principal investigator and represent 21 species in the genus. If differences among species in assortative preference are driven by geographic relationships, then sympatric species will have stronger assortative preference than allopatric species. We also compared the strength of assortative preference between males and females. Most sexual selection theory predicts that females will be choosier, however, given abundant evidence of male mate choice in darters, and the contrasting results of two studies of assortative mating, the relative importance of male and female mate choice in sexual isolation in darters remains an open question.

## 2 MATERIALS AND METHODS

### 2.1 Inclusion criteria

We conducted a literature search in the Web of Science database on March 8, 2024, with the following keywords in the TOPIC field: Etheostoma AND (mate choice OR mate prefer* OR behavio*). Of the 210 results obtained, 194 did not research mate choice, 17 researched mate choice but intraspecifically, 1 was a conference proceeding with no associated paper, and 16 studies corresponded to our criteria for researching mate choice by comparing preferences between conspecific and heterospecific individuals. Of those 16 studies, 3 allowed physical interactions between focal fish and reported proportions of measured behaviors (e.g., nosedigs and female pursuit) as a proxy for mate preferences (Moran et al., 2017; Moran & Fuller, 2018; Zhou et al., 2015), while the 13 other studies did not allow physical interactions and reported times spent in an association zone. Comparing the outcomes of mate choice studies that do and do not allow physical interactions between focal individuals could provide an important indication of the effect of these interactions on mate choice. In this case, however, because only three studies of one kind were available, and their metrics of choice (e.g., nosedigs by females in an open arena) are not obviously comparable to association time, we did not include them in the meta‐analysis.

In total, we included those 13 published papers that reported times spent in association zones (references provided in the Table S1) as well as four unpublished datasets (provided on OSF) with similar metrics, encompassing 21 species of *Etheostoma* distributed across the phylogeny of the genus (Near et al., 2011), arranged in 14 different pairs of species. We received four datasets from published papers after contacting the authors. The rest of the data were locally stored on hard disks or available online along with the published paper.

**FIGURE 1:**
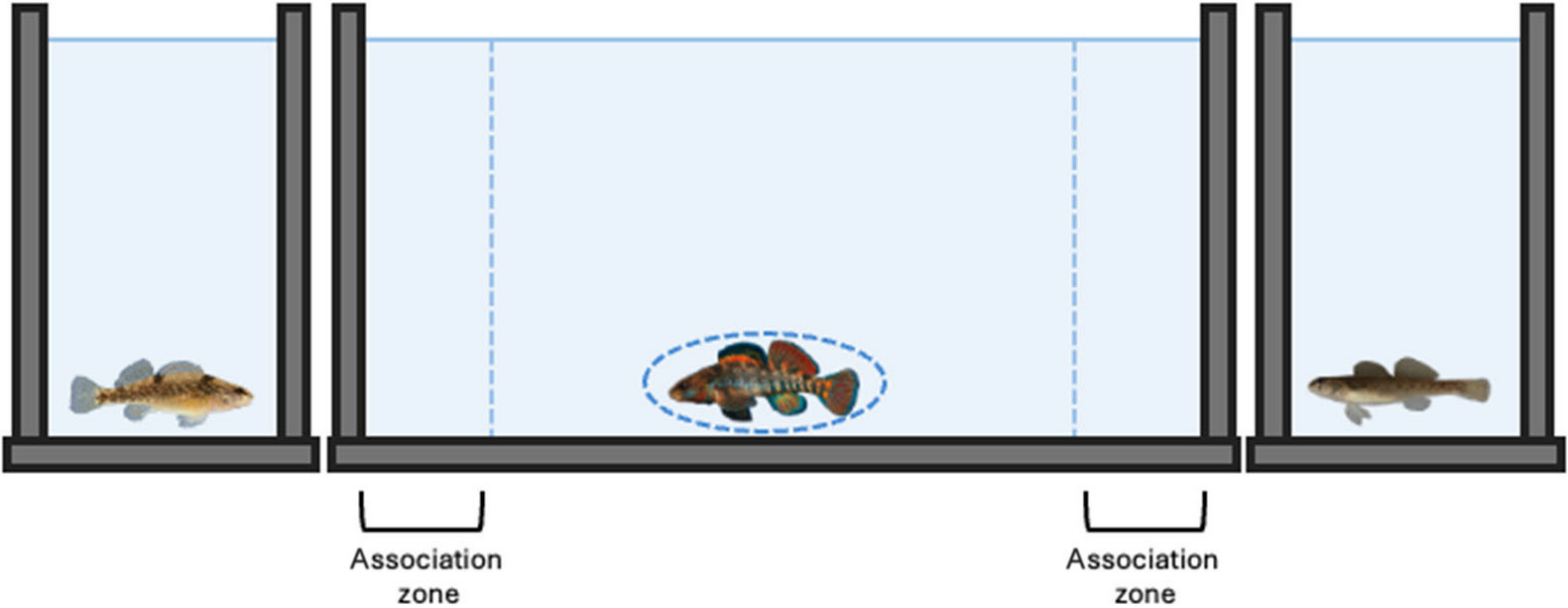
Mate preference experimental paradigm. Illustration of a dichotomous mate preference paradigm. The main measure is the time that the focal fish (circled) spends in either association zone adjacent to either a conspecific or a heterospecific individual of the opposite sex. The exact design varies between studies as mate options can be live fish, motorized models, videos, or computer animations displayed on a monitor.

All studies used a dichotomous mate preference paradigm where individuals do not have physical access to one another. The main measure of preference is the time the focal fish spent in an association zone adjacent to either a conspecific or a heterospecific individual (the “stimulus” fish) of the opposite sex, presented simultaneously (Figure 1). Most studies allowed only visual cues, with focal and stimulus fish separated into different tanks. Two studies (Barber, S. & Mendelson, T.C., unpublished; O'Rourke & Mendelson, 2010) used partitions that were not water tight to separate focal and stimuli fish, thus potentially allowing the exchange of olfactory cues. We limited our analysis to studies with this experimental design as it removes aggressive physical interactions among members of the same sex that can impede the expression of mate preference (see above). Studies in our analysis were conducted by different lead authors in different physical lab spaces but at the same university, using similar protocols.

### 2.2 Effect size calculation

All included studies measured the time spent in the association zones. Sample sizes, means, and standard deviations were extracted from each paper and when those variables were not available, we contacted corresponding authors to obtain the raw data. The effect size was calculated as Cohen's *d* for times spent in conspecific association zones minus heterospecific association zones for each tested species and sex of each study (Equation 1):

(1) $$d=\frac{{\underline{\mu }}_{\text{sampl}e_{C}}-{\underline{\mu }}_{\text{sampl}e_{H}}}{\sigma_{\text{pooled}}}$$

$$\text{with}\;\sigma_{\text{pooled}}=\frac{\sigma_{\text{sampl}e_{C}}2*\left(N_{\text{sampl}e_{C}}-1\right)+\sigma_{\text{sampl}e_{H}}2*\left(N_{\text{sampl}e_{H}}-1\right)}{N_{\text{sampl}e_{C}}+N_{\text{sampl}e_{H}}-2}$$

where ${\underline{\mu }}_{\text{sample}}$ and $\sigma_{\text{sample}}$ correspond to the mean and standard deviation of the time spent with conspecifics (${\underline{\mu }}_{\text{sampl}e_{C}}$) and with heterospecifics (${\underline{\mu }}_{\text{sampl}e_{H}}$), $N_{\text{sample}}$ corresponds to the sample size of tested individuals, which is identical for both conspecifics and heterospecifics in our case.

A positive effect size will correspond to preferences for conspecifics, while a negative effect size will correspond to preferences biased towards heterospecifics, and an effect size not different from 0 to an absence of assortative mate preference.

### 2.3 Moderators and their rationale

We sought to determine which factors might influence the strength of preference for conspecifics over heterospecifics, as represented by the effect size. We selected two “natural” factors: geography and sex of the tested individual, and three “experimental” factors: the size of the association zones, stimulus type, and recording duration times.

#### 2.3.1 Geography

We predicted the geographic relationship between the two species in a pair to influence the strength of preference for conspecifics, with a stronger preference for sympatric species. For each study, we determined whether species pairs were allopatric or sympatric (Etnier & Starnes, 1994; Lee et al., 1981; Page, 1983) and included this variable as a moderator. Some species pairs consist of both allopatric and sympatric populations (i.e., incomplete range overlap); pairs were scored according to the population of origin.

#### 2.3.2 Sex

Although classical sexual selection theory predicts a stronger preference in females, some studies have found the opposite pattern, with a stronger preference for conspecifics in males compared to females (e.g., Mendelson et al., 2018; Moran & Fuller, 2018). Our dataset includes as many male‐focal individuals as females, which allows us to compare assortative preferences between the sexes.

#### 2.3.3 Experimental factors

Previous work showed that experimental design impacts mating preference outcomes (Dougherty & Shuker, 2015). We thus included two moderators to reflect the variability in experimental setups. Namely, we included the size of the association zones (5 or 10 cm) and recording duration times (5, 10, 15, or 20 min) as experimental factors in our model. We predict that a larger association zone and longer recording duration will result in stronger effect sizes as more data are included. Note that for one of the included studies (Mendelson et al., 2018), the raw data included measures for times spent in association zones of both 5 and 10 cm. Because the statistical data reported in the paper were for the association zone size of 5 cm, we included effect sizes corresponding to that zone size in our meta‐analysis, after first using the raw data to confirm there was no difference in time spent between both association zone sizes, using a paired *t*‐test (*t* = 0.17255, df = 53.129, *p* = .8637).

### 2.4 Phylogeny

**FIGURE 2:**
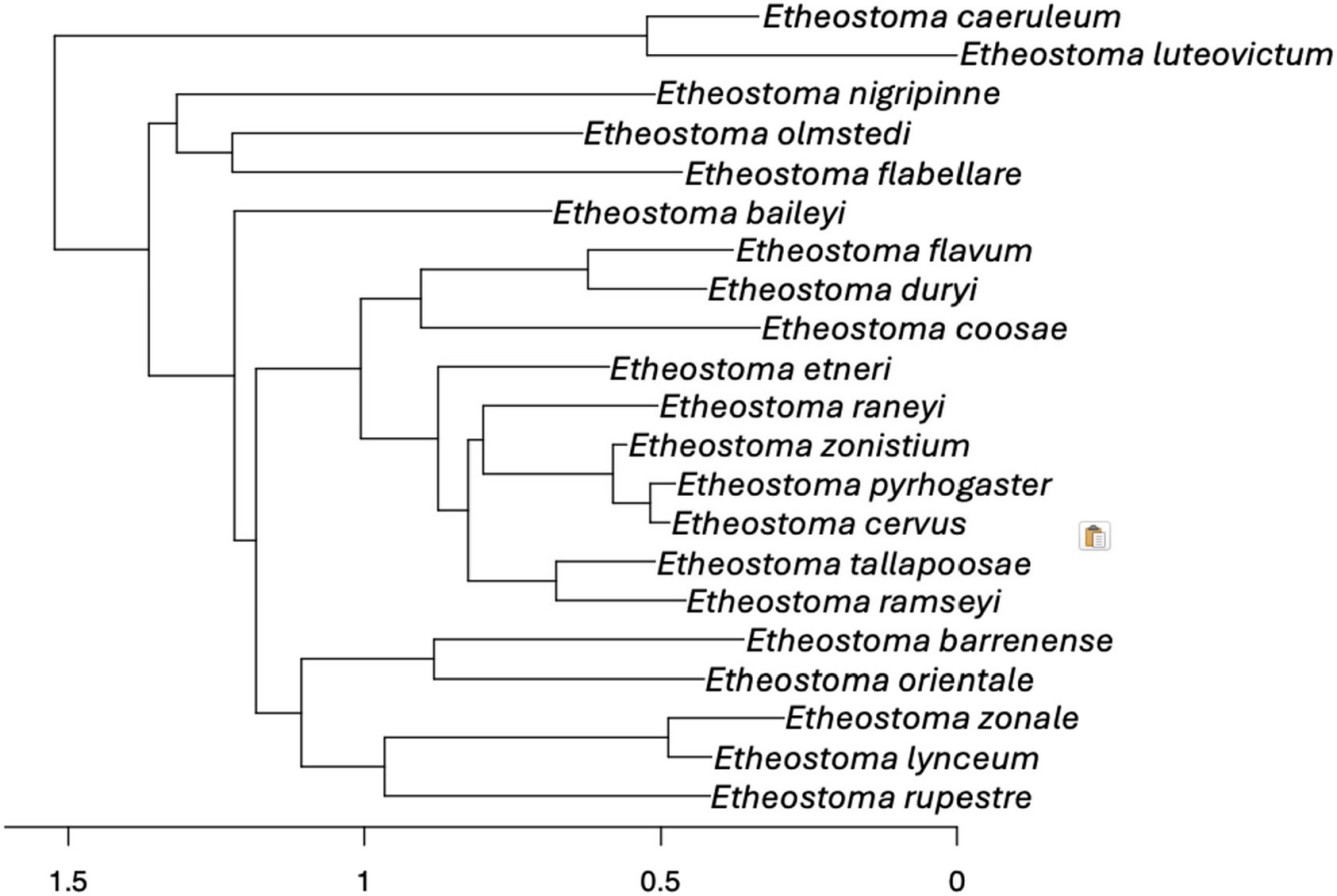
Phylogeny of the focal species included in the meta‐analysis. Phylogenetic tree of the 21 focal species included in the meta‐analysis based on the phylogeny of Near et al. (2011). The *x*‐axis is a unit‐free indication of time, with more recent species being on the right side.

To control for the non‐independence of the strength of preference due to a shared evolutionary ancestry that varies between species pairs, we included phylogenetic information in our statistical models, using Near et al.'s (2011) phylogeny. Their phylogeny is based on the cytochrome b mitochondrial gene and two nuclear gene sequences, the S7 ribosomal protein (first intron), and the recombination activating gene‐1 (RAG1, exon 3). We retrieved the data file (Nexus format) from TreeBase. We pruned the phylogenetic tree with the drop. tip function from the ape R package (Paradis & Schliep, 2019) to keep only our 21 species of interest. In the case where several individuals per species were available, we kept the individual from the closest geographical population to the population studied in our meta‐analysis. The resulting tree (Figure 2) was converted into a matrix of phylogenetic distance that was included in our meta‐regression models.

### 2.5 Statistical analyses

**FIGURE 3:**
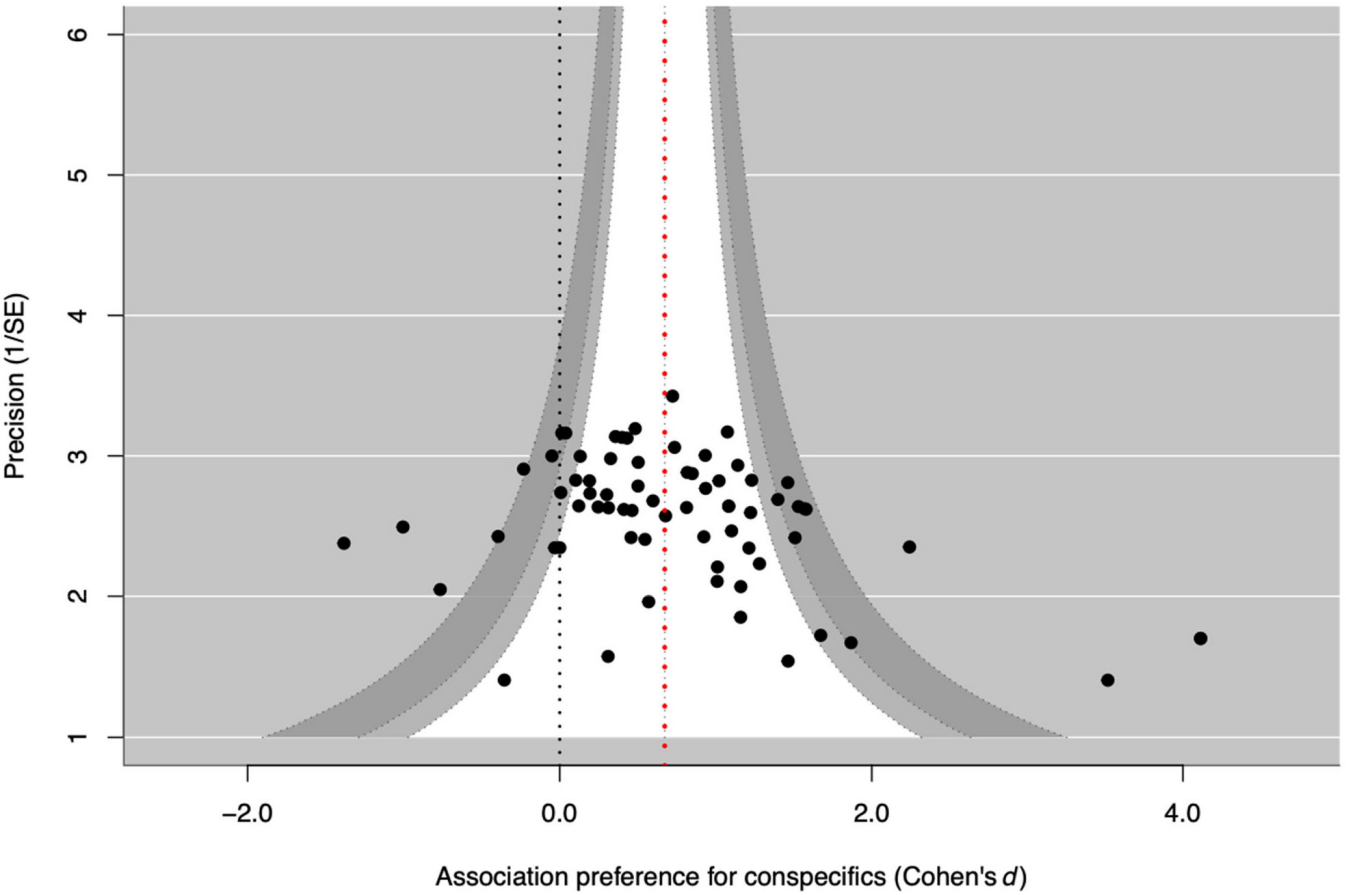
Funnel plot to test for publication bias. A funnel plot showing the 70 effect sizes extracted from 17 studies on 21 species. In the absence of publication bias and heterogeneity, 90% (white area), 95% (light gray area), and 99% (dark gray area) of studies would be expected to lie within the dotted confidence interval lines. The pooled estimate, or overall effect size (Cohen's *d*), shown as the red dotted line, is significantly bigger than zero (black dotted line). The funnel plot is asymmetrical, indicating some publication bias, which was confirmed by Egger's regression test: *Z* = 3.6939, *p* = .0002: Small and medium, positive effect sizes are overrepresented in the data.

All statistical analyses were carried out in R version 4.2.0 (R Core Team, 2022). We used the package metafor (Viechtbauer, 2010) to perform the meta‐analysis modeling. To determine the overall mean effect size, we ran a first multi‐level meta‐analysis model fitted via restricted maximum‐likelihood (“REML”) estimation with the function rma.mv. We included study identity as a random effect to account for the non‐independence of effect sizes. We removed species as an additional random effect as this variable explained 0% of the variance and removing it slightly decreased the AIC score of the model. Phylogeny was included in all our models as a variance–covariance matrix estimated from the phylogenetic tree. To assess the respective influence of our different moderators (i.e., explanatory factors) on the mean effect size, we ran meta‐regression models for each moderator separately (function rma.mv with the “mods” parameter). We calculated the level of heterogeneity across all effect sizes using the *I* ^2^ statistic to determine how generalizable our findings are (Higgins et al., 2003). The *I* ^2^ statistic is an estimate of the percentage of variability explained by between‐studies differences rather than by sampling error. We estimated the presence of a publication bias using Egger's regression test and by visually assessing the level of symmetry of a funnel plot (Figure 3). Egger's regression test is a linear regression of the effect estimates on their standard errors weighted by their inverse variance. In the absence of publication bias and heterogeneity, the majority of studies would be expected to lie within the dotted confidence interval lines, while the funnel plot should be roughly symmetrical and Egger's regression non‐significant.

Script for statistical analyses and data are available on OSF.

## 3 RESULTS

In total, we extracted 70 effect sizes from 17 studies investigating 21 focal species of darters. This includes 34 effect sizes for males and 36 for females, 42 for allopatric and 28 for sympatric populations.

Our multi‐level meta‐analysis model revealed an overall large (Cohen, 1998) and positive effect size (*d* = 0.8957, *p* < .0001, CI = 0.4747–1.3168), corresponding to positive preferences for conspecifics. The total heterogeneity across effect sizes (*I* ^2^) amounts to 79.81% (8.14% from the phylogeny, 39.36% from the study identity, and 32.31% from the effect size identity), revealing that we have highly heterogeneous effect sizes as data sampling variation only contributes to 20.19% of the total variation in effects.

A funnel plot showing the 70 effect sizes revealed the presence of a publication bias, which was confirmed by Egger's regression test: *Z* = 3.6939, *p* = .0002. Small and medium, positive effect sizes are overrepresented in the data.

**FIGURE 4:**
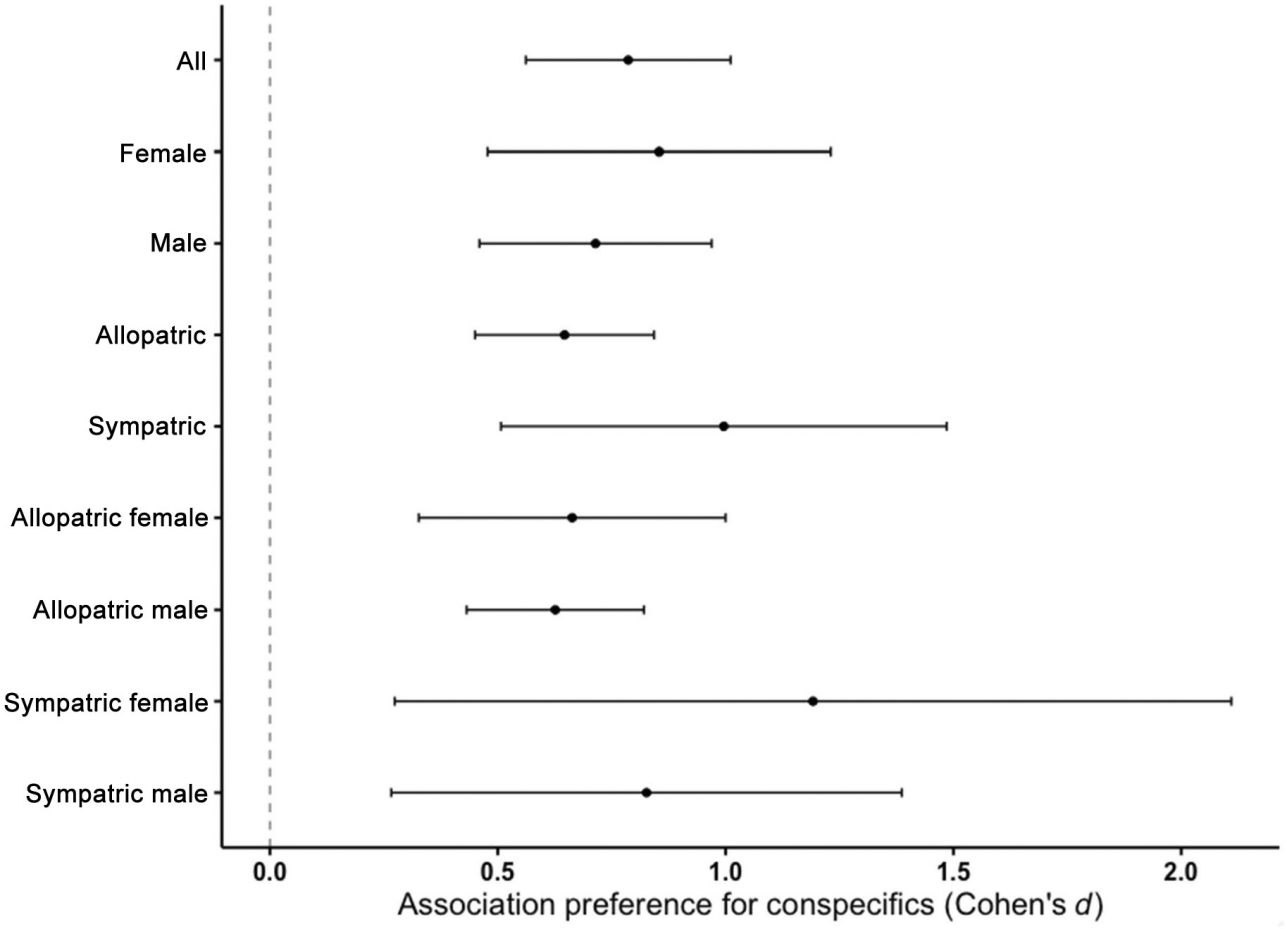
Forest plot of effect sizes. Forest plot showing mean effect sizes (± CIs) calculated using Equation 1 of the main moderators: sex and geographic relationship, as well as an average of all the moderators included in the analysis. Note that the male/female and allopatric/sympatric estimates come from separate meta‐regression models.

Our investigation of the respective influence of our moderators with separate meta‐regression models showed that none of them impacted variation in effect sizes. The results of our meta‐regression models are summarized in Table 1. We found no overall difference in preference strength between males and females and no difference between sympatric and allopatric populations as indicated in Figure 4. We also found no effect of the experimental factors we included (size of the association zones and recording duration times). However, given the highly unbalanced sample sizes for those experimental factors, conclusions should be carefully drawn.

**TABLE 1:** Table with moderators: *Q*_M_, *p*‐value, marginal *R* ^2^ (the amount of variance explained by each factor), mean, and CIs estimated in separate models for each moderator.

Moderator	*Q*_M_	*p*‐Value	Marginal *R* ^2^	Mean	Sample size	95% CIs
Sex of the focal individual^a^	0.3684	.5439	.0023	F: 0.3018 M: 0.3544	34 34	0.1104–0.4932 0.1572–0.5516
Allopatry (17 species) vs. sympatry (4 species)	1.1363	.2864	.0232	A: 0.2552 S: 0.3731	42 28	0.0515–0.4588 0.1410–0.6052
Size of the association zone	0.0515	.8204	.0020	5 cm: 0.2914 10 cm: 0.4002	59 11	0.0908–0.4920 0.1093–0.6912
Recording times (all) *Recording times (15 and 20 min only)*	0.9153 *(0.6026)*	.8217 *(0.4376)*	.0435 *(0.0393)*	5 min: 0.3991 10 min: 0.3789 15 min: 0.4397 20 min: 0.2686	4 3 19 44	−0.1316–0.9297 −0.2631–1.0209 0.2066–0.6728 0.0618–0.4755

^a^ Only data for species where both males and females were tested are included. This excludes *Etheostoma caeruleum* and *E. duryi*.

## 4 DISCUSSION

Our meta‐analysis of 17 datasets encompassing 21 species of darters revealed a positive mean effect size of preference for conspecific mates when using a dichotomous paradigm based on visual cues. This result suggests that most species included in our analysis prefer conspecifics of the opposite sex over heterospecifics based primarily on visual information. Our investigation of potential moderators of the strength of this preference suggests that neither geographic relationships (i.e., sympatry vs. allopatry), sex, nor any of the three experimental factors (association zone size, stimulus type, and recording time durations) predictably influence the strength of preference for conspecifics.

We investigated the effect of geographic overlap on the strength of assortative preference, as sympatry is thought to modulate assortative mating. Based on the findings of Coyne and Orr (1989) in *Drosophila*, whose estimation of prezygotic isolation for sympatric pairs was at least twice as large as for allopatric pairs, as well as other studies of single species pairs showing a similar pattern (Höbel & Gerhardt, 2003; Pauers & Grudnowski, 2022), we predicted stronger assortative preference in sympatric species of *Etheostoma*. Our statistical analyses did not support this prediction. Rather, of the four sympatric species included in the meta‐analysis, two showed a stronger preference for conspecifics (*E. zonale* and *E. barrenense*), one had no assortative preference (*E. flabellare*) and one, *E. olmstedi*, showed unexpectedly disassortative preference, with individuals from sympatric populations showing a stronger preference for heterospecifics (Figure S1). Interestingly, allopatric populations of *E. olmstedi* also included in the analysis did not show such a reverse pattern but rather a stronger preference for conspecifics (Figure S2).

Variation across species in the relationship between geographic overlap and assortative preference might occur if species, and populations within species, differ in the costs and benefits of mating with heterospecifics. The classic cost of mating with heterospecifics is a reduction in offspring fitness, and the strength of this postzygotic barrier is known to vary across species pairs (e.g., Martin & Mendelson, 2013; Mendelson, 2003). Alternatively, the variation we observed might reflect differences in the extent of syntopy between nominally sympatric species. For example, *E. barrenense* and *E. zonale*, a species pair in the analysis in which both species exhibit strong assortative preference, are notably syntopic where they overlap. *Etheostoma barrenense* is more strongly associated with bedrock substrate and *E. zonale* with vegetation (Greenberg, 1991; Hlohowskyj & Wissing, 1986), yet both are commonly found and collected together over bedrock or coarse gravel (pers obs). Given evidence of reduced survival in the hybrids of this species pair (Williams & Mendelson, 2014), assortative preference between these species may have arisen due to reinforcement (see Roberts & Mendelson, 2020). In contrast, *E. olmstedi* and *E. flabellare*, between which preference is either absent (*E. flabellare*) or disassortative (*E. olmstedi*), are known to exhibit distinct habitat and foraging preferences (Greenberg, 1991; Proulx, 2014) and may represent micro‐allopatric species. Thus, the conditions that predict stronger assortative preference in sympatry, including the opportunity for maladaptive hybridization, may not hold for this species pair.

In addition to geography, we investigated the effect of sex on the strength of preference for conspecifics. Classical sexual selection theory predicts that females will be choosier than males, as the cost of investment is expected to be skewed toward females (Andersson, 2004; Trivers, 1972). In our meta‐analysis analyzing male preference in 19 species and female preference in 21 species, sex did not have a significant effect on the strength of preference for conspecifics. Although counter to conventional sexual selection theory, this result is consistent with the natural history of darters. Male darters invest considerably in reproduction, with energetic courtship displays, nuptial coloration, and in some species, paternal care (e.g., Kelly et al., 2012; Mendelson et al., 2018). The absence of an effect of sex on assortative preference is also consistent with several previous studies in darters. For example, assortative preference is stronger in males than in females for allopatric species of darters in the earliest stages of divergence (Mendelson et al., 2018). Yet, in other studies, female darters exhibited stronger assortative preference than males, as in *E. nigripinne* (O'Rourke & Mendelson, 2010). Thus, the absence of an effect of sex on the strength of preference seems to accurately reflect the diversity of reproductive behaviors and preferences in this genus.

Additional factors that might generate variation among species in the strength of assortative preference, irrespective of geography, include the presence or absence of nuptial coloration. Assortative mating based on color pattern differences has been shown in several animal taxa, including Midas cichlid fish (Elmer et al., 2009), *Heliconius* butterflies (Jiggins et al., 2001), and strawberry poison frogs (e.g., Summers et al., 1999). Notably, two species in our analysis that show strong assortative preference are strikingly colorful, and previous studies show that both males and females of these species preferentially associate with conspecific color patterns (Williams & Mendelson, 2011, 2013). In contrast, the species in our analysis that lacks assortative preference (*E. flabellare*) and the species for which assortative preference varies across populations (*E. olmstedi*) are largely achromatic. Alternatively, species might differ in temporal patterns of mating, with some mating earlier or later in the season. Like micro‐allopatry, this type of temporal reproductive isolation prevents maladaptive hybridization and thus precludes selection against hybrids leading to increased assortative preference in sympatry.

Beyond the biological implications of our results, we also sought to determine whether experimental factors could influence effect sizes when comparing multiple studies. Although we did not expect a significant effect of recording time duration, we found a tendency for longer durations to have smaller effect sizes. It might thus be important to keep the duration of observation short (i.e., under 10 min) as the expression of preference may begin to taper after a few minutes as the focal individual loses interest. Besides mean durations in association zones, additional measures of fish preference could provide important information. Additional behavioral measures might include the fish's head orientation or line of sight, and pursuit behaviors, to quantify interest in the presented stimuli. For instance, two studies in darters reported glass jabbing behavior as a measure of a female's mating interest and of a male's aggressive behavior (Soudry et al., 2020; Williams & Mendelson, 2013). One study also reports the number of times a fish visits an area (Soudry et al., 2020), which could indicate exploratory differences between species or sexes that may reflect preference. The second experimental factor that varied between studies was the size of the association zones. Effect sizes tended to be larger for wider association zones, which is logical since a bigger area of the tank can be occupied for a longer period. To avoid inflating results with larger association zones, we recommend adjusting the size of the association zone to reflect the visual acuity of the tested species (Caves et al., 2017).

In our study, we restricted our analyses to studies in which all fish, stimulus and focal, were physically restrained from one another. Although this design is less realistic than allowing full contact, it can reveal preferences of the focal fish that might otherwise be masked by physical interactions or other behaviors of the stimuli. For example, males could physically compete with one another causing a preferred male to be excluded by a dominant but less preferred male. Alternatively, a nonpreferred stimulus fish may be overly solicitous or coercive, mating with the focal fish despite not being the preferred option. As to whether preferences in dichotomous mate choice trials are manifest in the natural conditions of the stream, there is a general correspondence in darters between preference strength as measured in dichotomous trials and in‐stream trials (Martin & Mendelson, 2013; Mendelson et al., 2018; Williams et al., 2013; Williams & Mendelson, 2010, 2011); any discrepancies in the strength of assortative preference in the two experimental designs could be accounted for by physical interactions. Thus, our analysis shows that quantifying preference without interference reveals an overall preference for conspecific mates.

In all but two of the studies in our analysis, focal and stimulus fish were exposed only to visual information. Darters appear to be highly visual stream fish (Gumm et al., 2012; Gumm & Mendelson, 2011; Williams & Mendelson, 2010), presumably attuned to the movement of macroinvertebrates in the stream and to the vivid color patterns of males. The proximate basis of sexual isolation, including its genetic, neural, sensory, and behavioral underpinnings, is a central question at the intersection of behavioral ecology and evolutionary biology (Davis et al., 2021; Gould et al., 2010; Rodríguez et al., 2004; Xu & Shaw, 2019), and our results indicate that assortative preference in the genus *Etheostoma* has a robust visual component.

*Etheostoma* is an emerging model for understanding the role of mate choice and sexual selection in speciation. Approximately 150 extant species comprise the lineage, which arose from a single common ancestor over the past ~30 million years (Near et al., 2011). Species in the genus exhibit diversity in morphology, habitat preferences, nuptial ornamentation, parental care, and spawning behavior, as well as community composition and structure, offering a rich opportunity to examine factors promoting speciation in a phylogenetic context. A growing number of studies examine mate choice in darters using a variety of experimental designs both within and between species. Our study highlights the need to consider interspecific and even population‐level variation when inferring general patterns in this system.

## 5 CONCLUSION

Conducting a meta‐analysis of dichotomous mate preference trials that measured time spent with conspecifics versus heterospecifics of the opposite sex in darter fish (*Etheostoma*), we found an overall effect size of medium strength, indicating assortative preference across the genus, with no significant difference between males and females. Comparing sympatric and allopatric populations, we found no effect of geographic overlap on the strength of assortative preference. All studies in our analysis prevented physical contact between focal and stimulus fish, and the vast majority allowed only visual information, suggesting that assortative preference in this genus is based at least in part on visual cues or signals. Our results suggest that the link between assortative mating and geography is not as straightforward as might be predicted from hypotheses of reinforcement and that the link between assortative mating and sex is not as straightforward as classical interpretations of sexual selection might suggest, at least in the studied genus. We postulate several factors that could modulate the strength of assortative preference in the genus *Etheostoma* and explain the lack of evidence for an overall effect of geography and sex. Our results thus highlight the complexity of assortative mating and the need for further study. Given the diversity of the genus, *Etheostoma* is well suited to investigate such questions.

## AUTHOR CONTRIBUTIONS

**Yseult Héjja‐Brichard:** Conceptualization (lead); data curation (lead); formal analysis (lead); investigation (lead); methodology (lead); resources (lead); software (lead); visualization (lead); writing – original draft (lead); writing – review and editing (lead). **Julien P. Renoult:** Methodology (supporting); supervision (supporting); validation (supporting); visualization (supporting); writing – original draft (supporting); writing – review and editing (supporting). **Tamra C. Mendelson:** Conceptualization (supporting); project administration (supporting); supervision (lead); validation (lead); visualization (supporting); writing – original draft (equal); writing – review and editing (equal).

## FUNDING INFORMATION

This work was supported by the National Science Foundation grant NSF IOS 2026334.

## CONFLICT OF INTEREST STATEMENT

None.

### OPEN RESEARCH BADGES

This article has earned an Open Data badge for making publicly available the digitally‐shareable data necessary to reproduce the reported results. The data is available at https://osf.io/hnf8m/.

## Supporting information


Data S1.

## Data Availability

Scripts for statistical analyses and data are publicly available (https://osf.io/hnf8m/).
