# Supplementary material for: Comparative analysis reveals assortative mate preferences in darters independent of sympatry and sex
Source: Ecol Evol. 2024 Sep 29;14(10):e11498. doi: 10.1002/ece3.11498 (PMC11439589; doi:10.1002/ece3.11498)
Supplement: Supplementary file 1 — Data S1. [file ECE3-14-e11498-s001.docx]

# Supplementary information


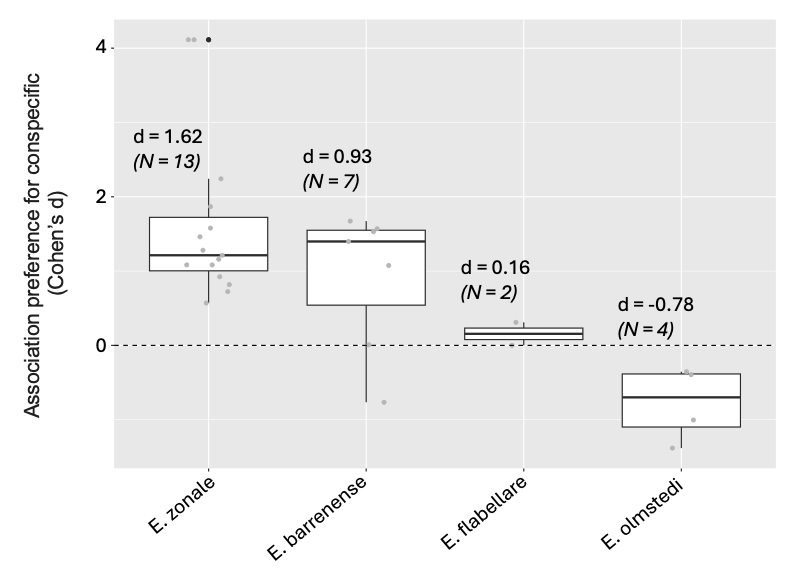


Figure S1: Effect sizes for the sympatric populations included in the meta-analysis (descending order). d gives the effect size value for the sympatric population of the species. N indicates the number of effect sizes (i.e. number of experiments) that were included per population. The grey jitter points represent the individual effect sizes.


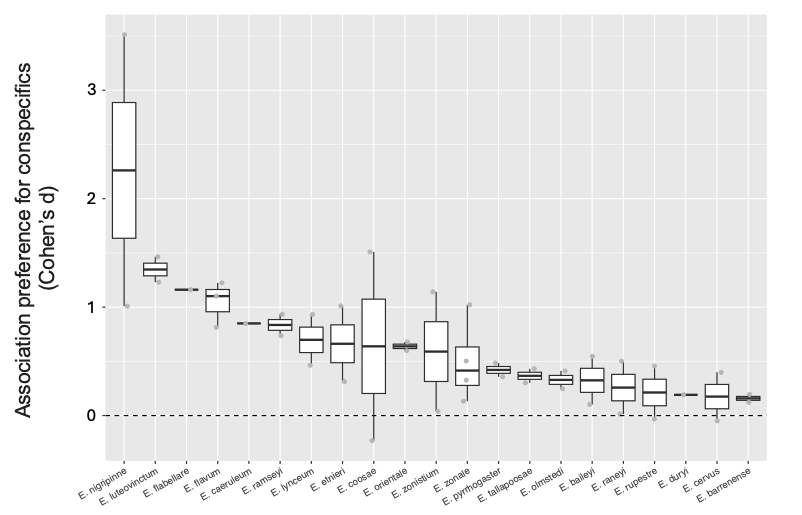


Figure S2: Effect sizes for the allopatric populations included in the meta-analysis (descending order).

Table S1: Studies included in the meta-analysis

| Study | Species tested | Number of effect sizes extracted |
| --- | --- | --- |
| O’Rourke and Mendelson (2010)  Williams and Mendelson (2010)  Williams and Mendelson (2011)  Cicotto et al. (2013)  Martin and Mendelson (2013)  Williams and Mendelson (2013)  Roberts et al. (2017)  Roberts and Mendelson (2017)  Mendelson et al. (2018)  Roberts et al. (2019)  Mattson et al. (2020)  Roberts and Mendelson (2020)  Roberts and Mendelson (2021)  Barber and Mendelson (unpublished)  Hulse and Mendelson (unpublished)  Taylor and Mendelson (unpublished)  Héjja-Brichard and Mendelson (unpublished) | *E. nigripinne*  *E. zonale*  *E. barrenense*  *E. zonale*  *E. barrenense*  *E. luteovinctum*  *E. flavum*  *E. duryi*  *E. zonale*  *E. barrenense*  *E. zonale*  *E. barrenense*  *E. zonale*  *E. barrenense*  *E. cervus*  *E. pyrrhogaster*  *E. ramseyi*  *E. tallapoosae*  *E. raneyi*  *E. zonistium*  *E. barrenense*  *E. orientale*  *E. lynceum*  *E. rupestre*  *E. baileyi*  *E. coosae*  *E. etnieri*  *E. flavum*  *E. zonale*  *E. barrenense*  *E. zonale*  *E. zonale*  *E. flabellare*  *E. caeruleum*  *E. olmstedi*  *E. olmstedi*  *E. flabellare* | 2  2  2  2  2  2  2  2  28  4  2  8  2  1  1  4  4 |
